# Opening up ZSM-5 Hierarchical Zeolite’s Porosity through Sequential Treatments for Improved Low-Density Polyethylene Cracking †

**DOI:** 10.3390/molecules25122878

**Published:** 2020-06-22

**Authors:** Karolina A. Tarach, Kamila Pyra, Kinga Góra-Marek

**Affiliations:** Faculty of Chemistry, Jagiellonian University in Kraków, 2 Gronostajowa St., 30–87 Kraków, Poland; kamila.pyra@doctoral.uj.edu.pl (K.P.); kinga.gora-marek@uj.edu.pl (K.G.-M.)

**Keywords:** hierarchical zeolites, acid-wash, Lewis acid sites, *operando* spectroscopy, LDPE cracking, realumination

## Abstract

An adequately tuned acid wash of hierarchical ZSM-5 zeolites offers a levelling up in the catalytic cracking of low-density polyethylene. Identification of crucial and limiting factors governing the activity of the zeolite was extended with studies about the accessibility of acid sites, nature of the realuminated layer and role of Lewis acid sites. The sequential treatment of a ZSM-5 zeolite offered enhanced activity in low-density polyethylene (LDPE) cracking at low and high conversions, as confirmed by a decrease in the temperatures needed to reach 20% and 80% conversion (T_20_ and T_80_, respectively). A linear dependence of the T_80_ on the coupled IHF (indexed hierarchy factor) and AF_B_ (accessibility factor) highlighted the importance of the textural and acidic parameters in the catalytic cracking of LDPE. *Operando* FT-IR-GC studies confirmed a higher fraction of short-chain hydrocarbons (C_3_–C_5_) in the product distribution of hierarchical catalysts resulting from the effective polymer cracking in easily accessible pores.

## 1. Introduction

Hierarchical zeolites are known to efficiently crack polymers or vacuum gas oil, giving high selectivity to most desirable products [1,2,3,4,5]. The benefits to these cracking reactions are the combination of a secondary mesopore system coupled with the intrinsic acidity of microporous zeolites [6,7]. The ZSM-5 zeolites are known to produce light olefins in the selective cracking of C_6_–C_9_ molecules; thus, they are expected to be an integral contributor to meeting the continuing global demand for light olefins [8]. The susceptibility of ZSM-5 zeolites to bottom-up modification has fuelled studies concerning the application of hierarchical ZSM-5 zeolites in cracking reactions [4,9]. Demetallation processes, i.e., desilication and dealumination, are highly versatile methods of hierarchical zeolite preparation; appropriately defined modification conditions ensure that hierarchical porosity can be obtained independently of Si/Al ratio, structure type or grains size [10,11]. Substantial interference in zeolite structure due to Si and Al atom extraction, however, leads to a significant alteration of the acid sites’ nature, preferentially with the formation of Lewis acid sites in a large number [12,13]. These Lewis acid sites are found in the majority on the newly created mesopores’ surface, as they originate from the Al-enriched layer formed during realumination process [13,14]. Although the acid sites’ accessibility increases upon desilication, the full potential of such hierarchical zeolites can be reached only by further precise treatment.

The improvement of the catalytic performance of zeolites containing EFAl (extra-framework Al) species originating from steaming or acid treatment in the cracking of alkanes remains a long-standing debate in zeolite science [8]. Many approaches have been considered, among them the possibility of EFAl being an active site for hydrocarbon activation, inductive influence of EFAl on Brønsted sites or enhancing acidity of Brønsted sites by withdrawing the electron density of neighbouring Si(OH)Al groups [8]. Nevertheless, it has also been shown that the removal of the Al-rich debris originating from desilication by subsequent acid treatment increases the activity of desilicated ZSM-5 zeolites for the alkylation of toluene with benzyl alcohol [10]. Additionally, extra-framework Al species formed via hydrolysis of framework Al perturbed or dislodged by desilication have been shown to restrict part of the channel structure of mordenite zeolite [15]. Their subsequent removal by mild acid leaching resulted in unrestricted channel openings and led to a large increase in the accessibility of Si(OH)Al groups for *n*-hexane. In contrast, the presence of unique Lewis acid sites formed by desilication enhances the isomerisation of dihydroxyacetone (DHA) to lactic acid (LA) [16]. The origin of these unique Lewis acid sites is still under debate. Generally, their formation is ascribed to the high susceptibility to dehydroxylation of the non-framework protonic sites of the realuminated layer [13,17]. The influence of the Lewis acid sites, and also of sequential treatments, on the catalytic activity have not been yet studied in polymer-cracking reactions.

Our previous study showed that a hierarchical ZSM-5 zeolite obtained by post-synthesis desilication was an effective catalyst for the cracking of low-density and high-density polyethylene (PE) polymers [4]. A linear correlation between the temperature necessary for reaching 50% PE conversion (*T*_50_) and acid sites accessibility indicated, with certainty, that an enhancement of mesopore surface area by hierarchisation led to an improvement in the *T*_50_ values. Nevertheless, for the hierarchical zeolite with the highest external surface area, the cracking activity was not improved as much as was expected by optimising the desilication conditions. That led us to the conclusion that the realuminated layer at the outer part of the zeolite grains may have been responsible for hindering the full potential of these zeolites in the PE-cracking reactions.

This work was devoted to proving the hypothesis of the negative influence of the realuminated layer on the polymer cracking. Sequential alkaline (deSi; desilication) and acidic (Ac; dealumination) treatments on a microporous ZSM-5 zeolite were performed, and the catalytic performance was verified in low-density polyethylene (LDPE) cracking. The comprehensive structural, textural and acidic characteristics of hierarchical zeolites were linked to their catalytic activity and selectivity.

## 2. Results and Discussion

**Figure 1:**
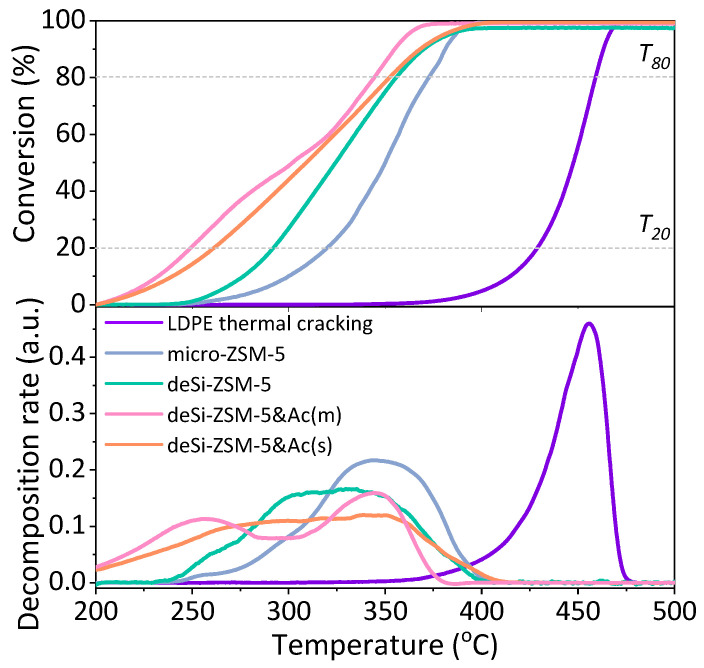
(**Upper**) LDPE (low-density polyethylene) catalytic cracking conversion curves (*T*_20_ and *T*_80_ the temperatures necessary for reaching 20% and 80% conversion) and (**lower**) decomposition rates of the studied zeolites as a function of temperature.

The catalytic LDPE cracking results showed that the zeolites modified in a sequential manner (deSi-ZSM-5&Ac(*)) were significantly more active in comparison to the desilicated cases (deSi-ZSM-5) (Figure 1). The *T*_50_ value was lowered from 325 °C to 300 °C for mild-acid-washed deSi-ZSM-5&Ac(m) compared with deSi-ZSM-5. Similarly, at the lower temperatures range, the *T*_20_ value shifted from 295 to 250 °C, giving the most pronounced enhancement of the cracking activity. However, only the carefully chosen conditions for the zeolite acid wash, deSi-ZSM-5&Ac(m), allowed this optimal activity to be achieved. Compared to the mild washing, when a more severe dealumination was applied, a decline of the activity was seen, as for the deSi-ZSM-5&Ac(s) sample. In general, the catalytic activity benefitted more from dealumination at reaction temperatures below 300 °C, and at higher temperatures, the acid-washed materials, particularly deSi-ZSM-5&Ac(s), resembled those that were desilicated only (*T*_80_ values differ slightly).

**Figure 2:**
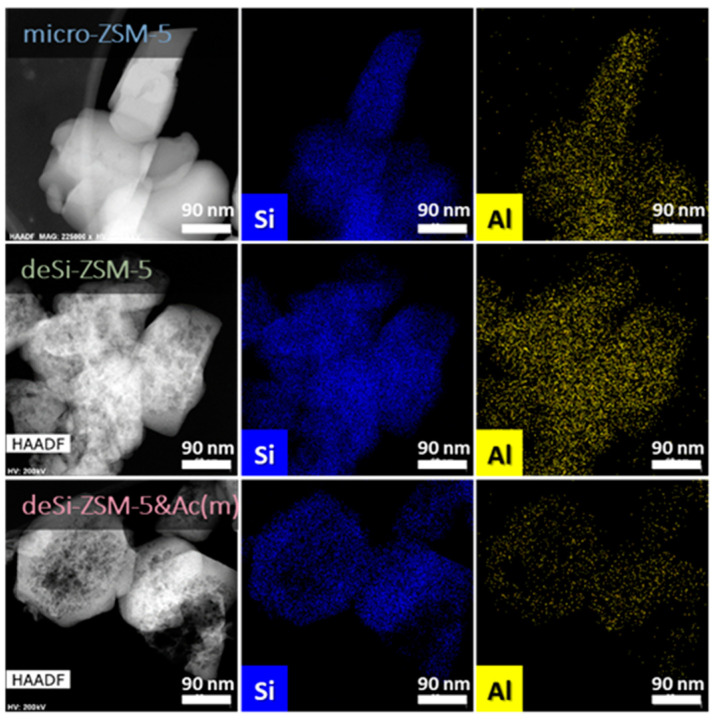
STEM images and EDX maps of Si and Al distribution in studied zeolites.

The enhancement in the catalytic activity of the modified zeolites, both desilicated and sequentially treated, can be explained by the upgraded textural and acidic properties. The increase of the mesoporous surface area upon desilication and related changes in the acidic properties enabled a higher catalytic activity. Furthermore, subsequent acid washing altered neither the structure nor the porosity (Table 1). The desilication itself led to the development of mesopore surface area (*S_meso_* = 146 m^2^g^−1^), accompanied only by a slight drop of the crystallinity (Cryst. = 84%, Supplementary Figure S1). The sequentially treated zeolites displayed crystallinity and porous characteristic parameters (*S_BET_*, *V_micro_*) similar to those of the deSi-ZSM-5. The acid washing did not affect the course of low-temperature N_2_ sorption isotherm, maintaining the mixed Type I/IV shape. The pore size distribution, with a broad range of mesopore sizes, was also preserved (Supplementary Figure S2). A fair indication of changes in the textural parameters was obtained via the indexed hierarchy factor (IHF, see Table 1) [10]. Higher IHF values point to a greater share of mesopores with preserved microporosity, while lower IHF values represent materials with excessive microporosity or mesoporosity. A comparable hierarchisation degree was quantitatively confirmed by the IHF values, ranging from 0.66 to 0.71. Visual proof of the formation of mesopores after desilication, and their preservation upon dealumination, was seen on STEM and TEM micrographs (Figure 2 and Supplementary Figure S3). All these textural findings indicated that the superior catalytic activity of the sequentially treated ZSM-5 zeolites should be related to their acidic features, being a derivative of Al content and speciation, since the crystallinity and texture did not change significantly with respect to the deSi-ZSM-5. Noticeable changes upon dealumination, compared with desilication were, however, seen in the chemical composition (ICP, Table 1) and Al distribution (EDX, FT-IR; Figure 2, Supplementary Figures S4 and S5) of the final materials. The expected drop of Si/Al after desilication (from 32 to 18) was followed by its increase for the sequentially treated deSi-ZSM-5-Ac(*) zeolites (from 18 to 22(m) or 25(s)). The leaching of Al from the zeolite surface grains in the dealumination process was also proved by EDX analysis. The Al atom distribution over the external surface of the zeolite grains upon dealumination (desi-ZSM-5&Ac(m)) was significantly altered (Figure 2).

The changes of chemical composition and Al distribution over the zeolite grains were reflected in the acidic properties (Table 2 and Supplementary Table S1).

The impact of the dealumination on the acidic properties of the deSi-ZSM-5 zeolite was examined comprehensively by probe molecule sorption in situ FT-IR spectroscopic studies. The detailed acidic feature assessment was performed with the use of pyridine (Py), pivalonitrile (Pn) and carbon monoxide (CO) to ascertain the total concentration, accessibility and nature of acid sites, respectively. The Py (Py_450_/Py_170_) and CO (Δν_CO…OH_) sorption results also served to assess the acid sites’ strength. Py as a probe molecule (kinetic diameter 0.54 nm) can penetrate the 10-ring channels of ZSM-5 zeolites and provide information on the total concentration of acid sites [20]. Pn is a branched molecule (kinetic diameter 0.69 nm) able to reach only the acid sites on the external surface of 10-ring zeolites and sites located near the pore entrances [19,21]; thus, it can be considered representative of LDPE molecules. The desilication led to an increase of the total concentration of acid sites (Supplementary Figures S4 and S5) due to the selective extraction of silicon species from the zeolite framework [4]. Upon subsequent dealumination, a predictable decrease in the number of Brønsted (B.a.s.) and Lewis (L.a.s.) acid sites was found.

As aforementioned, all the modified materials showed superior catalytic activity over the microporous counterpart. The following order of activity was found: micro-ZSM-5 < deSi-ZSM-5 < deSi-ZSM-5(s) < deSi-ZSM-5&Ac(m). The reason for the superiority of deSi-ZSM-5&Ac(m) was revealed through the scrutiny of the FT-IR spectroscopy results. Independently from the severity of dealumination, the ratios of B/L (Brønsted/Lewis) acid sites reached by Py (B/L_Py_) or Pn (B/L_Pn_) were constant (Table 2). Therefore, no differentiation between mild and severe acid washes in removal of the B and L acid sites was found. Nevertheless, the Al-originating species giving rise to L.a.s. in the deSi-ZSM-5 were predominantly removed over B.a.s. during the acid washing. The removal of this realuminated layer, detected as an excessive amount of L.a.s. in Py sorption, was the first feature directly related to the higher catalytic performance of both acid-washed deSi-ZSM-5&Ac(s) and deSi-ZSM-5&Ac(m). Upon dealumination, the acid strength was also preserved, in line with FT-IR results of Py and low-temperature CO sorption (Table 2). Furthermore, the alterations in the accessibility of B and L acid sites (expressed as B/L_Pn_ ratio) were simultaneous. What distinguished the mildly from the severely dealuminated sample was the explicitly expressed accessibility of B and L acid sites (Supplementary Figure S5). The accessibility factors [19] of Brønsted and Lewis acid sites (AF_B_ and AF_L_, resp.) were used for quantification (Table 2). Only mild dealumination ensured the highest accessibility of the acid sites and, as a result, the highest catalytic performance. Thus, the accessibility of sites is a second feature governing the catalytic activity. Nevertheless, the enhancement of the AF_B_ value for deSi-ZSM-5-Ac(m) was not accompanied by an increase of *S_meso_*, as is usually observed [4,19]. This implies that the accessibility of the acid sites was hindered by the presence of realuminated layer in deSi-ZSM-5. Thus, a comprehensive description of the zeolites’ hierarchisation degree when subsequent treatments are applied should consist of a dual factorisation by both the IHF and an acidic parameter, either B/L_Py_ or AF, presenting the full picture of acidity. The AF × IHF or B/LPy × IHF coupled factors are a proper measure of the changes accompanying a wide range of post-synthesis modifications and were later used to compare the zeolites’ performance in LDPE cracking. Besides the low accessibility of Brønsted acid sites, both desilicated and severely dealuminated samples were characterised by a declined accessibility of L.a.s. (AF_L_, Table 2). Thus, L.a.s. are supposed to be located in pore entrances or close to them, forming a realuminated layer, thus limiting the access for bulky Pn molecules. The overall accessibility of the acid sites should increase with Si/Al ratio [13]. However, the opposite finding for deSi-ZSM-5&Ac(s) implied a redistribution of aluminium in the zeolite framework disturbing the accessibility of acid sites.

**Figure 3:**
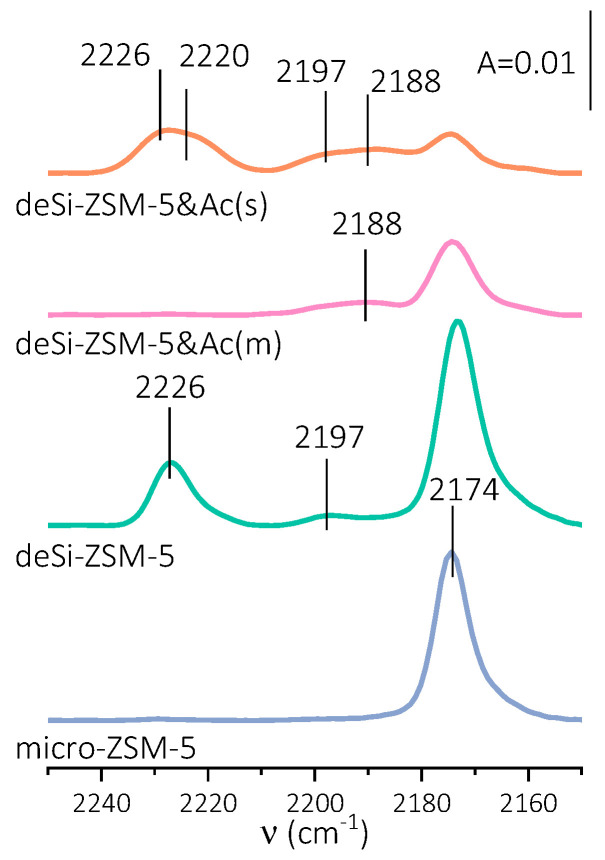
FT-IR spectra of CO sorbed at −100 °C onto zeolites after full saturation of Lewis acid sites, (for clarity, only spectra without full saturation of Brønsted acid sites are presented).

The preferential location of aluminium on the external surface was displayed by not only the declined accessibility of acid sites but mainly in the L.a.s speciation. Therefore, the nature of Lewis acid sites was identified as next, a third one, feature affecting the catalytic activity of the post-treated zeolites. Carbon monoxide is a probe molecule particularly suitable to discriminate the nature of L.a.s. Valuable information about the speciation and strength of Lewis acid sites can be attained from CO sorption, as the position of the CO band strongly depends on the electron-withdrawing properties of Lewis acid sites. Upon Py-thermodesorption studies, no distinct differences between the average strength of L.a.s. were found (Table 2). Therefore, to fully understand the alterations of the catalytic activity caused by the dealumination process, FT-IR studies of low-temperature CO sorption were undertaken (Figure 3). The spectra of the fully CO-saturated L.a.s. (2188, 2197, 2220 and 2226 cm^−1^ bands) demonstrated the dependence of their population on the applied post-treatment procedures. The IR spectra of CO adsorbed also displayed a band at 2174 cm^−1^ identified as CO hydrogen-bonded to Brønsted acid sites. The desilication (deSi-ZSM-5) led to a significant upsurge of strong L.a.s. from dehydroxylation (band at 2226 cm^−1^), which was accompanied by the appearance of weak Lewis acid sites (band at 2197 cm^−1^). In turn, a mild acid washing (deSi-ZSM-5&Ac(m)) ensured the total leaching of the strong L.a.s., but only slight changes in the electron-accepting character of weak L.a.s. were found (band downshifted to 2188 cm^−1^). Harsher dealumination conditions (desi-ZSM-5&Ac(s)) had an impact on L.a.s. characteristics, similarly to desilication treatment. The L.a.s. newly formed by the dehydroxylation were more heterogeneous, as manifested by two bands at 2226 and 2220 cm^−1^. Similarly, such heterogeneity is also observed for L.a.s. of weak electron-accepting properties (bands at 2197 and 2188 cm^−1^). After desilication, the appearance of strong Lewis acid sites from dehydroxylation seemed to be the main factor restricting the Brønsted acid sites’ accessibility (AF_B_, Table 2), and ultimately reducing the catalytic performance. The strong Lewis acid sites blocked micropore entrance, as was concluded from Pn sorption studies (Supplementary Figure S5, AF_L_ in Table 2) and, as a result, the accessibility of B.a.s. was significantly restricted. When properly adjusted dealumination conditions are applied, the hierarchical porosity becomes more open. The entrances to micropores from the mesopore surfaces were not clogged by Lewis residues longer, and were thus free of additional constraints. Harsher dealumination conditions suppressed the accessibility of acid sites; again, L.a.s. from dehydroxylation were found.

**Figure 4:**
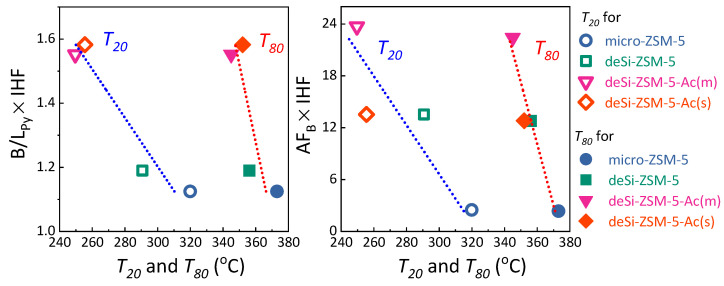
(**left**) B/L_Py_ × IHF and (**right**) AF_B_ × IHF coupled factors (B/L_Py_—ratio of B.a.s. to L.a.s; IHF—indexed hierarchy factor; AF_B_—accessibility factor of B.a.s.) plotted against the temperatures for 20% and 80% conversion (*T*_20_ and *T*_80_) for LDPE cracking.

However, the electron-acceptor properties of the L.a.s. in the dealuminated materials differed from those formed after desilication: the less electron-acceptor sites (of lower strength) were more populated in both acid-leached materials and, most importantly, the overall L.a.s. concentration was significantly lowered (Table 2, Supplementary Figure S5). Consequently, the impact of L.a.s. on the catalytic performance was less negative and deSi-ZSM-5&Ac(s) was still more active than deSi-ZSM-5 in the low-temperature range. It seems that the high concentration and strength of Lewis acid sites in deSi-ZSM-5 zeolite are responsible for restricted accessibility to Brønsted acid sites, what negatively affected the catalytic performance. The CO adsorption results gave a significant insight into the speciation of Lewis acid sites, nevertheless to fully understand the nature of the realuminated layer, the Py sorption data were needed. The Py sorption proved that Al-species giving rise to L.a.s. in deSi-ZSM-5 finally formed the realuminated layer. Its removal led to an increase of B/L_Py_ ratios and enhanced catalytic activity for both acid-washed samples. To highlight the influence of the realuminated layer on LDPE-cracking activity, the B/L_Py_ ratio, altered upon its formation (desilication) and subsequent removal (acid-washing), was coupled with IHF. Then B/L_Py_ × IHF coupled factors were plotted against the *T*_20_ and *T*_80_ values (Figure 4, left). The linear dependence confirmed that both the outer Lewis sites’ etching and hierarchical porosity played a significant role in the LDPE catalytic cracking. To further unwrap the impact of the three identified features on the catalytic activity, the AF_B_ × IHF coupled factors were also plotted against the *T*_20_ and *T*_80_ values (Figure 4). The deSi-ZSM-5&Ac(s) and deSi-ZSM-5 samples displayed almost identical AF_B_ × IHF values; nevertheless, at lower conversion, the former outperformed the latter. At this stage, at low temperatures, the polymer might be cracked only at the weak acid sites. The most stable carbocations were cracked at the highest temperatures, or, in other words, in the presence of the strongest sites. The two-step nature of the cracking process over zeolites was also well represented in the decomposition LDPE rates (Figure 1). At higher conversion (*T*_80_, Figure 4), when the preliminary cracking was realized over the external surface, the accessibility of B.a.s. played a major role and the impact of the realuminated layer negligible. Thus, the conversion curves for deSi-ZSM-5 and deSi-ZSM-5&Ac(s) showed a similar course (Figure 1). This points to the importance of realuminated-layer removal after desilication. For deSi-ZSM-5, both the high L.a.s. share in the overall acidity and low B.a.s accessibility negatively affected the catalytic performance at lower conversion values (*T*_20_). The L.a.s. in excessive amount were proved to quench the LDPE cracking over desilicated ITQ-4 at low conversion (10%), to a degree found for its microporous counterpart [22]. The deSi-ZSM-5&Ac(s) being free of realuminated layer was able to keep up with deSi-ZSM-5&Ac(m) activity only in the low conversion range; later on, the limited accessibility forced a drop of activity to the levels observed for deSi-ZSM-5. On the other hand, the deSi-ZSM-5&Ac(m) outperformed other zeolites as it combined the best feature, i.e., the highest AF_B_ which was not suppressed by L.a.s. originating from either dehydroxylation or realuminated layer.

**Figure 5:**
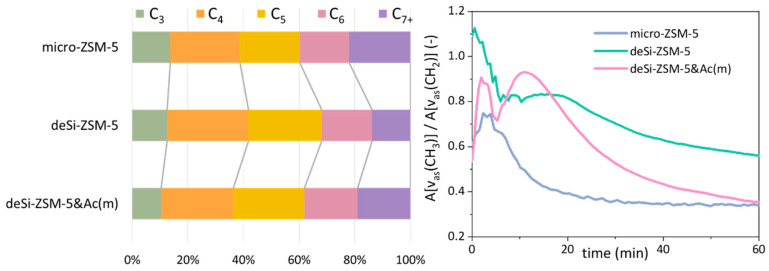
(**left**) Selectivity to C_n_ groups of hydrocarbons in total amount of products formed upon cracking of LDPE; (**right**) CH_3_/CH_2_ band intensity ratio derived from FT-IR *operando* studies as a function of time during LDPE cracking for microporous (micro), desilicated (deSi) and mildly acid-washed (Ac(m)) ZSM-5 zeolites.

The LDPE cracking over the studied catalysts was followed by FT-IR *operando* studies with on-line coupled GC analysis of the resulting products. The reaction temperature was adjusted to avoid the input from thermal cracking, i.e., 230 °C was applied. Two sets of information were provided by the FT-IR-GC *operando* studies (Figure 5). The quantitative analysis of the overall reaction products showed that deSi-ZSM-5 and deSi-ZSM-5&Ac(m) increased the selectivity to value-added light C_3_–C_5_ hydrocarbons at the expense of heavier C_7+_ fraction. This effect was more pronounced for the deSi-ZSM-5 catalyst, where a higher concentration of B.a.s. ensured a higher share of light hydrocarbon production. The deSi-ZSM-5&Ac(m) catalyst delivered a C_7+_ fraction in lower amounts than the micro-ZSM-5 catalysts (19% to 22%); nevertheless, the share of C_3_–C_5_ was comparable. This implied that the concentration of Brønsted acid sites dominated the selectivity of the LDPE-cracking reaction, while both acidity and hierarchical porosity governed the overall activity by means of *T*_20_ and *T*_80_ (Figure 1). An additional insight was provided by scrutiny of the bands representative for CH_3_ (2960 cm^−1^) and CH_2_ (2925 cm^−1^) groups. The ratio of CH_3_/CH_2_ band intensities was used to evaluate the susceptibility of PE end-chain cracking over catalysts (Figure 5). For micro-ZSM-5, the ratio increased in the first 3 min, reaching 0.75 as maximum, and then a successive drop was seen. Two-step curves are displayed for the hierarchical catalysts, which implied, similarly to the conversion curves (Figure 1), a dual nature of the cracking process. The end-chain cracking dominated for microporous zeolite because LDPE chains must get inside the channels, where they are cracked on strong acid sites. For desilicated zeolites, after some time middle-chain cracking began to dominate, which may suggest that the development of mesoporosity allowed easier diffusion and thus middle-chain cracking. Further increase in accessibility resulted in more noticeable middle-chain cracking. A slightly higher value of the CH_3_/CH_2_ ratio throughout the whole reaction time for the deSi-ZSM-5 than for deSi-ZSM-5&Ac(m) catalyst was reflected in the increased share of C_3_–C_5_ hydrocarbons in the product distribution. After preliminary cracking, during the first 5 min of reaction, the observed trends corresponded well with the AF_B_ × IHF coupled factors. However, at longer time-spans in isothermal conditions, the concentration of B.a.s. seemed to play crucial role in cracking of LDPE, as the deSi-ZSM-5&Ac(m) catalysts resembled the micro-ZSM-5.

## 3. Conclusions

A careful dealumination procedure leads to the effective redistribution of Al species onto the mesopore surface. Such a procedure, therefore, offers the highest possible accessibility of the acid sites. The micropore entrances become free of the restrictions from blocking by strong Lewis residues. To the best of our knowledge, such a correlation between the selection of dealumination conditions and the restricted accessibility of the Brønsted acid influencing polymer-cracking activity is reported here for the first time. Three co-dependent features ruling the cracking activity of hierarchical ZSM-5 zeolites were identified. Their impacts were rationalised both in low and high conversion ranges of LDPE cracking. These correlations, and the importance of the acid sites’ accessibility, were directly reflected in the catalytic performance and could serve as a prerequisite for catalysts’ applicability. The FT-IR-GC *operando* studies proved that the selectivity of LDPE cracking in isothermal conditions is ruled by catalyst’s acidic feature. The surface of deSi-ZSM-5&Ac(m) catalysts free of Al-realuminated ensured the highest catalytic performance. The temperature needed to achieve the highest conversion was significantly reduced while the selectivity for value-added C_3_–C_5_ hydrocarbons was maintained.

## 4. Materials and Methods

### 4.1. Materials

The micro-ZSM-5 zeolite (NH_4_-form, Zeolyst, Kansas, MO, USA, CBV 5524G) was transformed to the H-form by calcination at 550 °C for 5 h with a rate of 2 °C/min. The desilication procedure was carried out with the use of a 0.2 M solution of NaOH at a temperature of 80 °C for 0.5 h. After desilication, the mixture was put into an ice bath, filtered and washed thoroughly with distilled water until neutral pH was reached. Next, a fourfold ion-exchange with the use of a 0.5 M NH_4_NO_3_ solution was performed (60 °C, 1 h). The obtained sample was filtrated, washed and dried at room temperature. The dealumination treatments were then applied at a temperature of 80 °C for 1 h. Mild acid washes were perfumed with the use of a 0.05 M solution of HNO_3_, while the severe acid washing was done with 0.10 M of the same acid. After the acid treatment, the samples were filtrated, washed and dried at room temperature. All modified samples were calcined at 550 °C for 5 h, at a rate of 2 °C/min.

### 4.2. Characterisation of the Catalysts

The chemical composition was assessed by inductively coupled plasma–optical emission spectroscopy (ICP-OES, PerkinElmer, Krakow, Poland, Optima 2100DV). The measurements were performed to determine Si/Al molar ratios of the parent and modified samples. The sample, typically 80–100 mg, was digested in a Teflon vessel with the use of HCl (4 cm^3^, 35%) and HF (0.4 cm^3^, 48%). The final concentration was adjusted with deionised water.

Wide-angle powder X-ray diffraction (XRD) patterns were obtained using a Rigaku Multiflex diffractometer equipped with Cu Kα radiation (40 kV, 40 mA). The relative crystallinity was assessed by comparing the integrated area of the characteristic reflections of the MFI type structure in the range of 22.5° to 25.0°. The crystallinity of the calcined micro-ZSM-5 sample was referenced as 100%.

The porosity of the materials was studied by low-temperature adsorption/desorption of nitrogen. The isotherms were obtained from a Quantachrome Autosorb-1-MP gas sorption analyser. Before each measurement, the samples were heated at 350 °C and evacuated under high-vacuum conditions (ca. 10^−5^ mbar) for 16 h. The micropore volume (*V_micro_*) and surface area of micropores (*S_micro_*) were calculated using the *t*-plot method. The specific surface area (*S_BET_*) was calculated based on the Brunauer–Emmet–Teller (BET) method, following the recommendations of Rouquerol et al. [18,23]. The pore size distributions were obtained from the adsorption isotherm branch using the Barrett–Joyner–Halenda (BJH) model [24].

The electron micrographs were obtained in a JEOL 2100F transmission electron microscope working at 200 kV, equipped with a field emission gun (FEG). EDX and STEM detectors were used, working under bright and dark modes.

The FT-IR studies with the use of probe molecules were performed on Vertex 70 spectrometer (Bruker, Poznan Poland) equipped with an MCT detector. The applied resolution was set at 2 cm^−1^. The spectra for quantitative analysis were normalised to the same sample mass (10 mg). The study was performed with the use of pyridine (Py, ≥99.8%, Sigma-Aldrich, Grajewo, Poland), pivalonitrile (Pn, 98%, Sigma-Aldrich, Grajewo, Poland) and carbon monoxide (CO, Linde Gas Poland, 99.95%) as probe molecules. Before the analysis, the samples were pressed into self-supporting discs (5–10 mg·cm^−2^) and pre-treated in situ at 500 °C under high-vacuum (10^−6^ mbar) conditions for 1 h.

*Py sorption:* Py vapours (>20 Tr) in an amount sufficient to neutralise all acid sites were introduced into the FT-IR cell at 170 °C. Next, to remove the gaseous and physisorbed Py, evacuation at the same temperature was performed. This was confirmed by the disappearing of the respective Py bands. The concentration of acid sites was estimated based on the respective bands’ intensities (band height) and their absorption coefficients (1545 cm^−1^, PyH^+^, 0.07 cm^2^·µmol^−1^ and the 1450 cm^−1^, PyL, 0.10 cm^2^·µmol^−1^) [20,25]. The acid strength was determined in thermodesorption experiments. The preservation of the 1545 cm^−1^ band (PyH^+^) and 1455 cm^−1^ (PyL) upon desorption at 450 °C was estimated. The ratios of PyH^+^_450_/PyH^+^_170_ and PyL_450_/PyL_170_ were then taken as measures of the acid strength of Brønsted and Lewis sites, respectively.

*Pn sorption:* Pn sorption in an excess amount was done at room temperature. The gaseous and physisorbed molecules were then removed over 20 min evacuation at the same temperature. The concentration of the sites accessible to the Pn molecules was estimated based on respective bands intensities (band height) and their absorption coefficients (2277 cm^−1^, Pn…H^+^, 0.11 cm^2^·µmol^−1^ and 2305 cm^−1^, Pn…L, 0.15 cm^2^·µmol^−1^) [19].

*CO sorption*: The sorption of CO was performed at −100 °C. The Lewis acid sites’ nature was studied in experiments involving sorption of small doses of carbon monoxide. The total saturation of Lewis sites was monitored as the maximum intensities of the bands in the region of 2300–2180 cm^−1^. The total saturation of Lewis sites was also documented by the appearance of the Si(OH)Al···CO band at 2174 cm^−1^, as Brønsted sites are able to ligate CO molecules in the next order due their lower electron-accepting properties compared to Lewis sites. The dosing of CO was then increased, and the total saturation of Brønsted acid sites was observed. The downshift (Δν_CO…OH_) of the Si(OH)Al···CO band of the adducts was taken as a measure of the acid strength.

### 4.3. Catalytic Cracking of Low-Density Polyethylene

Low-density polyethylene (LDPE, ≤400 microns) from Alfa Aesar (Haverhill, MA, USA, Product No.: 42607, Lot No.: P28D047) was used for the catalytic tests. Thermogravimetric analysis was performed using a TGA/SDTA Mettler Toledo apparatus. The polymer (30 mg) and the zeolite powder (10 mg) were thoroughly mixed in an agate mortar for 10 min. A certain portion of the prepared fresh mixture (ca. 10 mg) was then transferred into an α-Al_2_O_3_ crucible. During the analysis, a flow of nitrogen was supplied (80 mL/min), and the temperature was raised from 30 °C to 600 °C at a heating rate of 5 °C/min. The catalyst weight and the adsorbed water content were taken into account when calculating the conversion. The conversion was defined as the weight loss of the mixed LDPE and zeolite sample, taking into account the content of water in the zeolite sample. The coke amount was measured by separate TG measurement where the weight loss of the spent catalyst upon heating to 800 °C was checked. The amount of non-volatile products (not exceeding 3.7% for each sample) was included in percentage of conversion.

### 4.4. Operando FT-IR-GC Studies of LDPE Cracking

The LDPE cracking was studied in an *operando* system (Supplementary Figure S6) connected to a flow set-up with nitrogen as a carrier gas (30 mL/min). The zeolites were mixed with LDPE (1:1), and pressed into self-supporting wafers (ca. 5.5–6 mg/cm^2^). The wafer was placed in a bespoke 2 cm^3^-volume IR quartz gas cell. The reaction cell allowed for the analysis of the gas phase and surface of catalysts simultaneously during the reaction. The reagents were transferred by 1/16” Teflon lines, heat-traced at 110 °C. Time-resolved FT-IR spectra were taken on a Vertex 70 Bruker FT-IR spectrometer (resolution 2 cm^−1^) over 60 min of reaction. The catalyst wafer in the cell was rapidly heated from room temperature up to 230 °C at 10 °C/s ramping rate. The decomposition of LDPE was performed at 230 °C. The reaction products were analysed by gas chromatography (Agilent 7890B).

## Figures and Tables

**Table 1:** Chemical composition, crystallinity and textural parameters of studied zeolites.

	Si/Al ^a^	Cryst. ^b^ %	*S_BET_*^c^ m^2^g^−1^	*S_meso_*^d^ m^2^g^−1^	*V_micro_*^e^ cm^3^g^−1^	IHF ^f^
micro-ZSM-5	32	100	377	40	0.17	0.21
deSi-ZSM-5	18	84	517	146	0.16	0.71
deSi-ZSM-5&Ac(m)	22	83	501	144	0.15	0.66
deSi-ZSM-5&Ac(s)	25	78	499	148	0.15	0.68

^a^ Concentrations of Si and Al from chemical analysis (ICP), expressed as Si/Al ratio. ^b^ Calculated based on XRD patterns in the range of 22.5° to 25.0°. ^c^ Calculated via BET method with the recommendations of Rouquerol et al. [18] ^d^ Calculated as the difference between *S_BET_* and *S_micro_*. ^e^ Calculated via the *t*-plot method. ^f^ IHF (indexed hierarchy factor) calculated as (*V_micro_*/*V_micro_*,_max_) × (*S_meso_*/*S_meso_*,_max_) [10]; *V_micro_*_,max_ and *S_meso_*_,max_ based on Reference [4].

**Table 2:** Acid sites properties derived from FT-IR studies of Py, Pn and CO sorption.

	B/L_Py_^a^	B/L_Pn_^b^	PyH^+^_450_/PyH^+^_170_^a^	PyL_450_/PyL_170_^a^	Δν_CO…OH_^c^ cm^−1^	AF_B_^d^ %	AF_L_^d^ %
micro-ZSM-5	11.3	7.9	0.94	0.99	315	12	17
deSi-ZSM-5	3.5	2.4	0.84	0.91	306	19	28
deSi-ZSM-5&Ac(m)	4.9	3.5	0.84	0.98	303	36	50
deSi-ZSM-5&Ac(s)	4.9	3.6	0.83	0.98	304	20	28

^a^ from Py adsorption: the ratio of Brønsted (B) and Lewis (L) acid site concentrations (B/L_Py_); the strength of Brønsted acid sites represented the PyH^+^ and Lewis acid sites (PyL) preserved upon desorption at 450 °C, PyH^+^_450_/PyH^+^_170_ and PyL_450_/PyL_170_, resp. ^b^ from Pn adsorption: the ratio of Brønsted (B) and Lewis (L) acid site concentrations (B/L_Pn_) accessible to bulky Pn molecules. ^c^ from CO sorption: the shift of Si(OH)Al group engaged in hydrogen bonding with CO molecules represents the acid strength of sites. ^d^ Accessibility factors of Brønsted (AF_B_) and Lewis (AF_L_) acid sites, i.e., shared (expressed in %) acid sites accessible to bulky Pn concerning their total concentration (Py) [19].

## Supplementary Materials

The following are available online, Figure S1. XRD patterns for all studied samples. Figure S2. N_2_ adsorption/desorption isotherms and pore size distributions for all studied samples. Figure S3. TEM micrographs (left), Si and Al EDX maps (middle) and Si, Al and HAADF micrographs (right) of studied samples. Figure S4. FT-IR spectra of pyridine interacting with the studied catalysts at 170 °C. Figure S5. Total concentration (full-colour) and share of accessible (pattern) Brønsted (B.a.s.) and Lewis (L.a.s.) acid sites from FT-IR studies of Py and Pn sorption versus Al content (grey line) from chemical analysis for all studied zeolites. Table S1 Acid sites properties derived from FT-IR studies of Py sorption. Figure S6. Real impression of the *Operando* reactor, showing the main sections. The rig allows feeding either gases or vapors, or both, using independent thermo-stated vapor-phase saturators. The mechanical parts are Teflon^TM^ based that guarantee working under inert conditions and avoiding cross-contamination. The Reactor cell is a Transmission cell with a rapid heating. This allows to obtain quantitative data for kinetic or deactivation studies. Finally, the gas outlet can be analysed by a gas chromatograph, FT-IR gas cell or mass spectrometers that are connected on-line.
