# Supplementary material for: Opening up ZSM-5 Hierarchical Zeolite’s Porosity through Sequential Treatments for Improved Low-Density Polyethylene Cracking
Source: Molecules. 2020 Jun 22;25(12):2878. doi: 10.3390/molecules25122878 (PMC7356772; doi:10.3390/molecules25122878)
Supplement: Supplementary file 1 [file molecules-25-02878-s001.pdf]

Electronic Supplementary Information

Opening up ZSM-5 hierarchical zeolite's porosity through sequential treatments for improved low-density polyethylene cracking

Karolina A. Tarach,* Kamila Pyra, Kinga Góra-Marek

Faculty of Chemistry, Jagiellonian University in Kraków,
2 Gronostajowa St., 30-387 Kraków, Poland

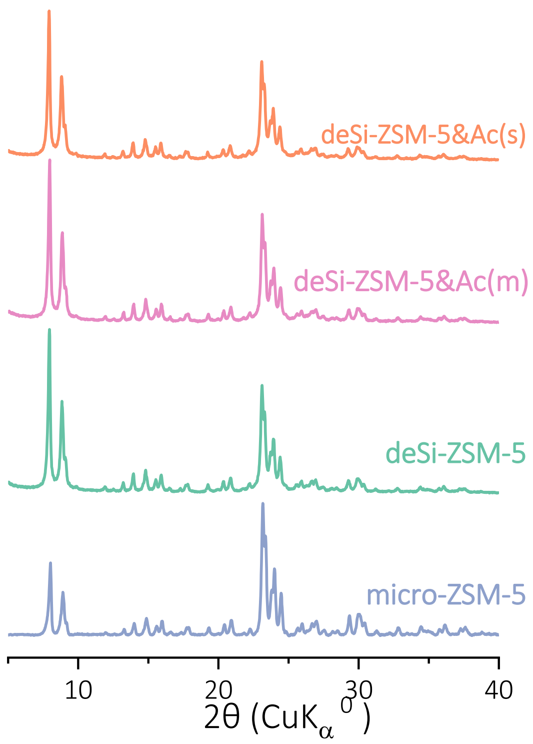

Figure S1. XRD patterns for all studied samples.

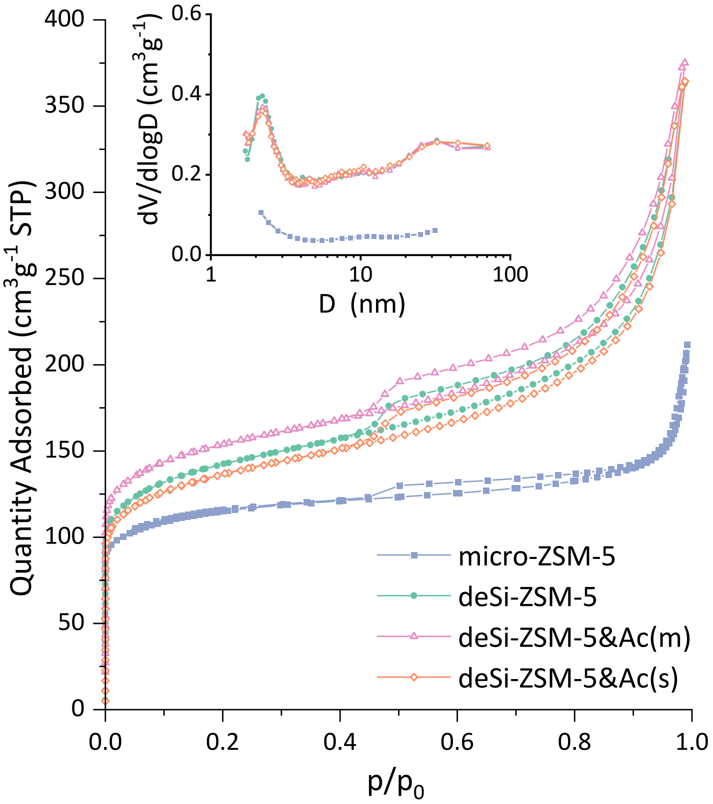

Figure S2. N_2 adsorption/desorption isotherms and pore size distributions for all studied samples.

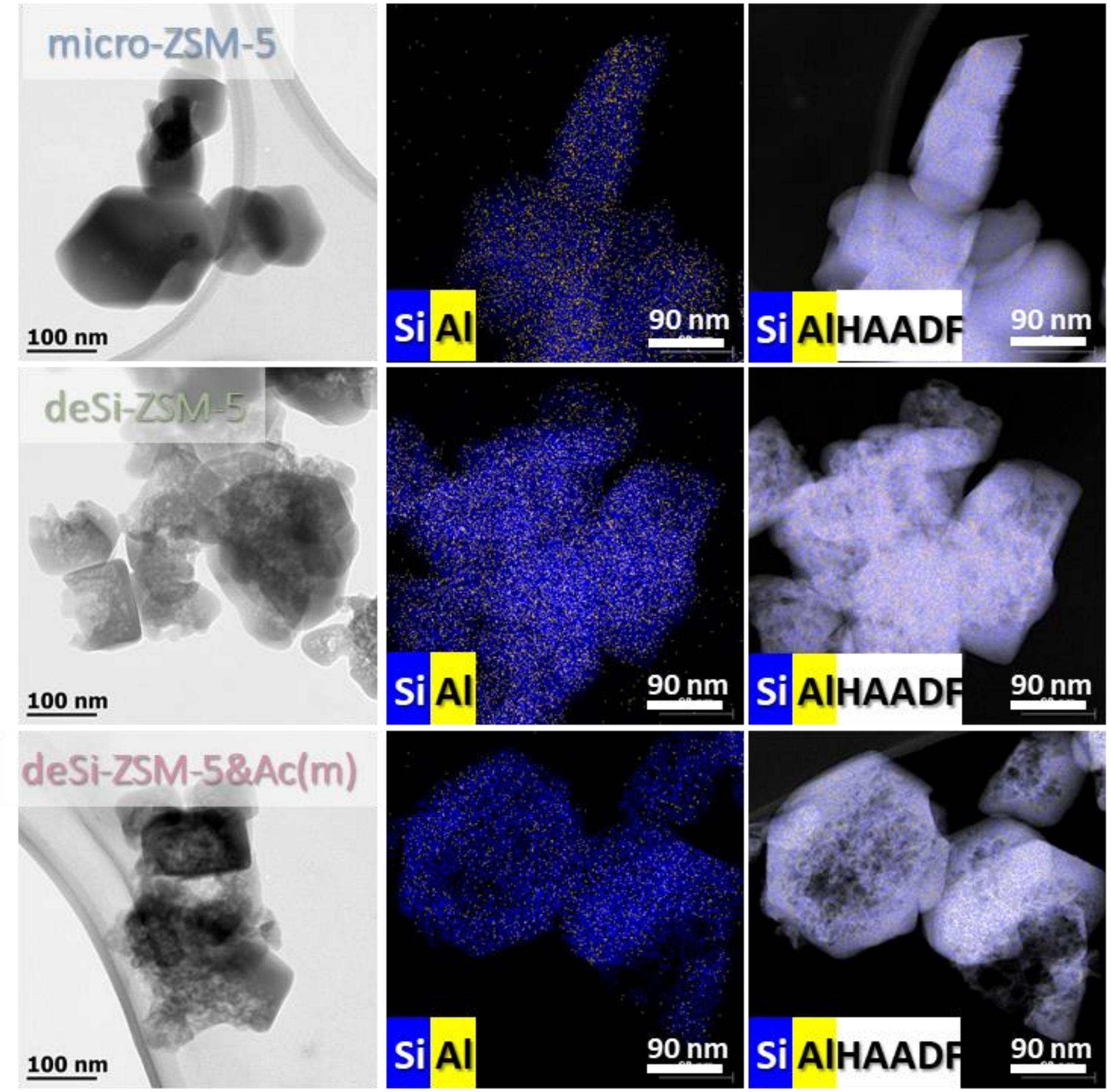

Figure S3. TEM micrographs (left), Si and Al EDX maps (middle) and Si, Al and HAADF micrographs (right) of studied samples.

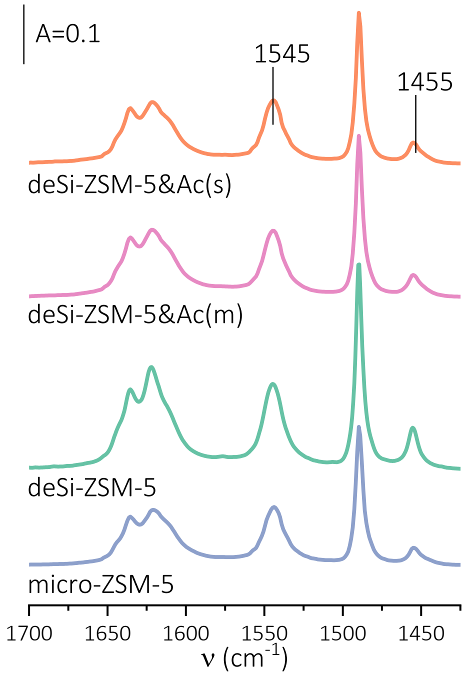

Figure S4. FT-IR spectra of pyridine interacting with the studied catalysts at 170 ⁰C.

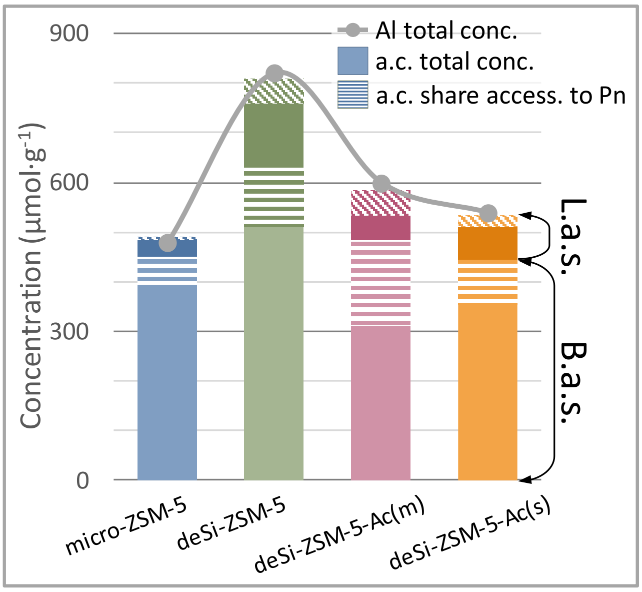

Figure S5. Total concentration (full-colour) and share of accessible (pattern) Brønsted (B.a.s.) and Lewis (L.a.s.) acid sites from FT-IR studies of Py and Pn sorption versus Al content (grey line) from chemical analysis for all studied zeolites.

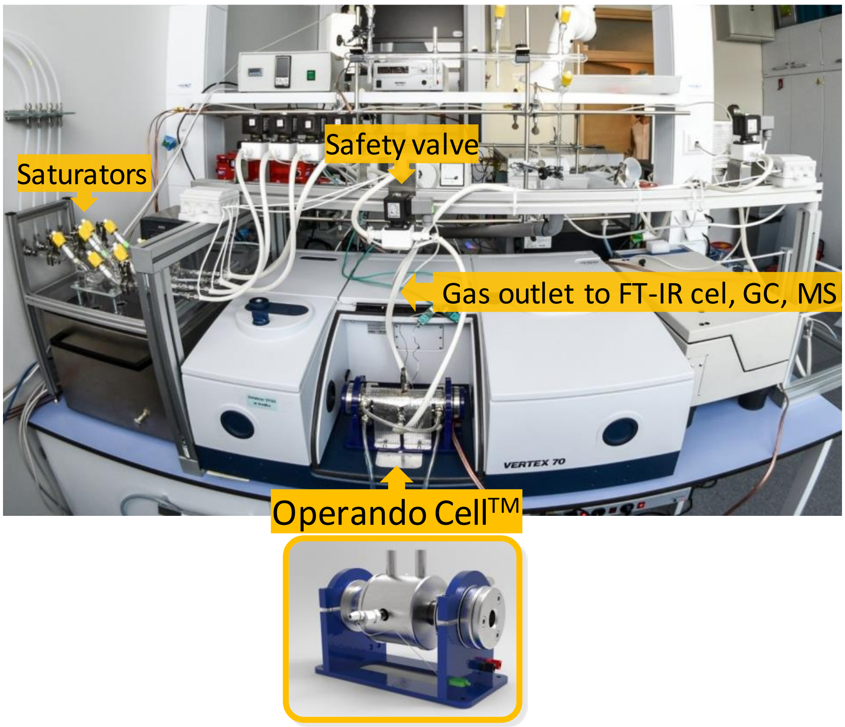

Figure S6. Real impression of the Operando reactor, showing the main sections. The rig allows feeding either gases or vapors, or both, using independent thermo-stated vapor-phase saturators. The mechanical parts are Teflon™ based that guarantee working under inert conditions and avoiding cross-contamination. The Reactor cell is a Transmission cell with a rapid heating. This allows to obtain quantitative data for kinetic or deactivation studies. Finally, the gas outlet can be analyzed by a gas chromatograph, FT-IR gas cell or mass spectrometers that are connected on-line.

Table S1 Acid sites properties derived from FT-IR studies of Py sorption.

	Si/Al[a]	Al[a] $\mu mol \cdot g^{-1}$	B.a.s. (Py) $\mu mol \cdot g^{-1}$	L.a.s. (Py) $\mu mol \cdot g^{-1}$	(B + L) a.s. (Py) $\mu mol \cdot g^{-1}$
micro-ZSM-5	32	479	443	41	484
deSi-ZSM-5	18	816	633	182	815
deSi-ZSM-5&Ac(m)	22	603	484	98	582
deSi-ZSM-5&Ac(s)	25	540	445	90	535

[a] concentration of Si and Al from chemical analysis (ICP) and expressed as Si/Al ratio or Al content

[a] from pyridine adsorption: the Brønsted (B) and Lewis (L) acid sites concentrations
